# Complete Genome Sequence of *Enterococcus hirae* R17, a Daptomycin-Resistant Bacterium Isolated from Retail Pork in China

**DOI:** 10.1128/genomeA.00605-16

**Published:** 2016-06-23

**Authors:** Zixin Peng, Wei Wang, Yujie Hu, Fengqin Li

**Affiliations:** Key Laboratory of Food Safety Risk Assessment, Ministry of Health, China National Center for Food Safety Risk Assessment, Beijing, The People's Republic of China

## Abstract

Daptomycin-resistant *Enterococcus hirae* R17 was isolated from retail pork sold at a free-trade market in Beijing, China. The complete genome sequence of R17 contains a circular 2,886,481-bp chromosome and a circular 73,574-bp plasmid. Genes involved in cell envelope homeostasis of this bacterium were identified by whole-genome analysis.

## GENOME ANNOUNCEMENT

Enterococci have tremendous genome plasticity and a propensity for increased transmission and dissemination of resistance elements through the food chain or environmental medium (1). The rise of multidrug-resistant enterococci, especially vancomycin-resistant enterococci (VRE), in the last few decades has led to challenges in clinical treatment (2). Daptomycin (DAP) is a lipopeptide antibiotic frequently used as a last-resort antibiotic against VRE infection (1, 3). However, one of the major problems when using DAP against enterococci is the emergence of resistance during therapy (4, 5). Previous studies report that mutations in regulatory systems involved in cell envelope homeostasis are postulated to be important mediators of DAP resistance in *Enterococcus faecium* and *Enterococcus faecalis* (6). DAP-resistant *Enterococcus hirae* R17 was isolated from retail pork sold at a free-trade market in Beijing, China. Human infection with *E. hirae* appears to be exceedingly rare, based on published reports, but all such cases involved bacteremia with severe illness (7). Therefore, the complete genome sequence of strain R17 was carried out in order to provide the genetic basis for DAP resistance mechanisms of *E. hirae* in the future.

Whole-genome sequencing of *E. hirae* R17 was performed using Pacific Biosciences RS II sequencing platform (Pacific Biosciences, Menlo Park, CA, USA). A 10-kb SMRTbell library was prepared from sheared genomic DNA using a 10-kb template library preparation workflow. Single-molecule real-time (SMRT) sequencing was conducted using the C4 sequencing chemistry and P6 polymerase with 1 SMRT cell. *De novo* assembly of the PacBio read sequences was carried out using continuous long reads (CLR), according to the Hierarchical Genome Assembly Process (HGAP) workflow (PacBioDevNet; Pacific Biosciences), as available in SMRT Analysis version 2.3 (8). The complete genome sequence of *E. hirae* R17 contains a circular 2,886,481-bp chromosome and a circular 73,574-bp plasmid (designated plasmid pRZ1), with G+C contents of 36.96% and 35.57%, respectively. There are 2,580 predicted genes in the chromosome in total, including 2,499 protein-coding genes, 63 tRNA-coding genes, and 18 rRNA-coding genes. There are 83 predicted protein-coding genes in plasmid pRZ1.

The functions of the predicted proteins were annotated based on homologs in a comparison to the NCBI-nr, Pfam, and KEGG databases. It was found, of all the proteins in *E. hirae* R17, 1,904 proteins have homologues in the evolutionary genealogy of genes: non-supervised orthologous groups (eggNOG) databases and assigned proper terms. The remaining have no orthologous groups (e-value < 1e - 5).

Genes related to biogenesis of cell wall/membrane/envelope and cell motility have been annotated in the genome sequence. These genes or gene clusters will further explain their potential relevance in DAP resistance. Virulence genes and antibiotic genes were also predicted by Virulence Factor Database and Antibiotic Resistance Genes Database, respectively. In conclusion, the genome of *E. hirae* R17 will enrich the DAP resistance genome database and facilitate the study of DAP resistance mechanisms.

### Nucleotide sequence accession numbers.

The complete genome sequence of *E. hirae* R17 has been deposited at the GenBank under the accession numbers CP015516 (chromosome) and CP015517 (plasmid pRZ1).
